# Deep learning downscaled high-resolution daily near surface meteorological datasets over East Asia

**DOI:** 10.1038/s41597-023-02805-9

**Published:** 2023-12-12

**Authors:** Hai Lin, Jianping Tang, Shuyu Wang, Shuguang Wang, Guangtao Dong

**Affiliations:** 1 Key Laboratory of Mesoscale Severe Weather/Ministry of Education, Nanjing University, Nanjing, 210023 China; 2 School of Atmospheric Sciences, Nanjing University, Nanjing, 210023 China; 3 Key Laboratory of Citie’s Mitigation and Adaptation to Climate Change in Shanghai, China Meteorological Administration, Shanghai, 200030 China

**Keywords:** Projection and prediction, Hydrology

## Abstract

U-Net, a deep-learning convolutional neural network, is used to downscale coarse meteorological data. Based on 19 models from the Coupled Model Intercomparison Project Phase 6 and the Multi-Source Weather (MSWX) dataset, bias correction and UNet downscaling approaches are used to develop high resolution dataset over the East Asian region, referred to as Climate Change for East Asia with Bias corrected UNet Dataset (CLIMEA-BCUD). CLIMEA-BCUD provides nine meteorological variables including 2-m air temperature, 2-m daily maximum air temperature, 2-m daily minimum air temperature, precipitation, 10-m wind speed, 2-m relative humidity, 2-m specific humidity, downward shortwave radiation and downward longwave radiation with 0.1° horizontal resolution at daily intervals over the historical period of 1950–2014 and three future scenarios (SSP1-2.6, SSP2-4.5 and SSP5-8.5) of 2015–2100. Validation against MSWX indicates that CLIMEA-BCUD shows reasonable performance in terms of climatology, and it is capable of simulating seasonal cycles and future changes well. It is suggested that CLIMEA-BCUD can promote the application of deep learning in climate research in the areas of climate change, hydrology, etc.

## Background & Summary

Climate change exerts tremendous influence on water resources^1,2^, agriculture^3^, and renewable energy^4^, particularly in densely populated regions like East Asia. In the context of global warming, water availability and agriculture are affected by increasing extreme events including floods^5^. Extreme temperature and precipitation not only pose risks to people’s safety but also inflict damage on agriculture crops throughout East Asia. On the other hand, climate change significantly affects the reliability and performance of the energy system, notably solar energy and wind energy^6^. Consequently, there is a growing emphasis on the assessment of climate change in East Asia within the broader context of global warming.

Global Climate Model (GCM) is a crucial tool for understanding climate change, as it can produce long-term and gridded climate information. However, due to the coarse resolution, GCMs are unable to represent the physical processes at fine resolution^7^. Moreover, due to limited knowledge of the earth system and simplified parameterisation, significant biases exist in GCM outputs in comparison to observations^8–10^. One way to remedy this is bias correction (BC) and downscaling, which are often considered as an essential step for the assessment of climate change. Bias correction is a statistic approach to reducing the discrepancy between GCM simulations and observations^11^. Downscaling technology aims to obtain data at finer resolution to characterize local-scale features. Usually, downscaling can be classified by dynamical downscaling (DD) and statistic downscaling (SD). Based on BC, SD approach can reduce the bias in GCM^12^, especially in CMIP6^13^. There are many downscaled datasets such as NEX-DCP30^14^ (0.5°), MACAv2-METDATA^15^ (2.5°), MACAv2-LIVNEH^16^ (3.75°), NEX-GDDP-CMIP6^17^ (0.25°) and Bias-corrected CMIP6 global dataset for dynamical downscaling of the Earth’s historical and future climate^18^ (1.25°). However, few downscaling datasets cover large-scale regions with a high resolution of 0.1° from CMIP6 under global warming.

SD establishes statistical relationships between large-scale GCM outputs at coarse resolution and local-scale observations at fine resolution during the training period, and applies the relationships to obtain fine information during the projected period. It is computationally inexpensive and easy to implement^19^. There are different SD approaches, for example, regression, weather classifications, and weather generators. Regression approaches are very popular, such as multi linear regression (MLR)^20^, generalized linear model (GLM)^21^, and machine learning (ML) method including support vector machine (SVM)^22^, random forests (RF)^23^, and artificial neural networks (ANN)^24,25^. Many studies have compared the performance among different regression approaches^26–29^.

Deep learning (DL) has been proved to be good at capturing complex and abstract features from numerous data^30^. Many studies have applied the DL based super-resolution (SR) approaches for downscaling^31–33^. Among the DL approaches, UNet shows superior performance in the field of SR and has been used in statistical downscaling. Sha *et al*.^34,35^ developed new UNet archives, named UNet-AE and Nest-UNet for temperature and precipitation downscaling respectively, and found that the UNet-based models show better performance than spatial disaggregation. Adewoyin *et al*.^36^ applied Temporal Recurrent UNet (TRU-NET) to downscale precipitation, and showed TRU-NET had better performance than a DL model prevalent in precipitation downscaling and dynamical downscaling method.

In this study, we develop a new bias correction and downscaling approach, named BC-UNet, to construct a Climate Change for East Asia with Bias Corrected UNet Dataset (CLIMEA-BCUD) based on CMIP6. The BC-UNet downscaling approach firstly applied Quantile Delta Mapping (QDM) to correct CMIP6 models biases based on the MSWX^37^ dataset at 1.0° × 1.0° spacing resolution^38^, then the UNet is trained for downscaling the biased corrected GCM dataset. The BC-UNet archive is applied to the historical simulations (1950–2014) and three future (2015–2100) scenarios of SSP1-2.6, SSP2-4.5 and SSP5-8.5. There are nine near-surface meteorological variables including 2-m air temperature (tas), 2-m daily maximum air temperature (tasmax), 2-m daily minimum air temperature (tasmin), precipitation (pr), 10-m wind speed (sfcWind), downward longwave radiation (rlds), downward shortwave radiation (rsds), 2-m relative humidity (hurs) and 2-m specific humidity (huss) (Table 1). CLIMEA-BCUD provides high-resolution large-scale DL downscaling in East Asia, which we suggest will be helpful for assessing climate change under global warming.

**Table 1:** Variables included in the CLIMEA-BCUD.

Variable	Description	Units
tas	Near-surface air temperature	°C
tasmax	Maximum near-surface air temperature	°C
tasmin	Minimum near-surface air temperature	°C
pr	Precipitation	mm/day
sfcWind	Surface wind speed	m/s
rlds	Surface downwelling longwave radiation	W/m^2^
rsds	Surface downwelling shortwave radiation	W/m^2^
hurs	Near-surface relative humidity	percentage
huss	Near-surface specific humidity	Kg/kg

## Methods

### Data acquisition

**Fig. 1:**
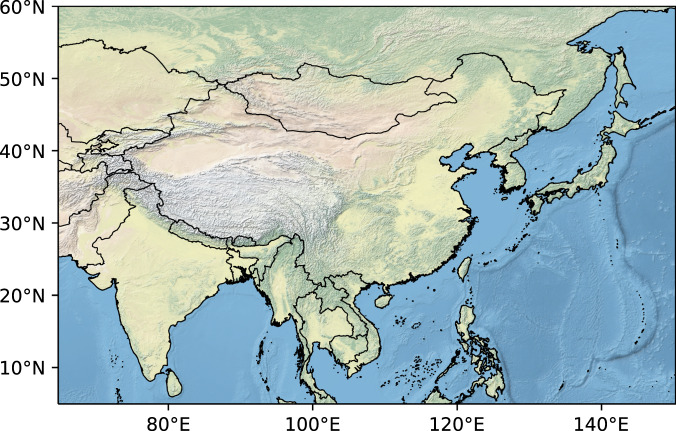
Study area in CLIMEA-BCUD.

The MSWX gridded high-resolution bias-corrected meteorological dataset is used as observations. Based on ERA5, MSWX produces 10 widely used near-surface meteorological variables with 0.1° horizontal resolution and 3-hour temporal resolution. The study area covers the whole of East Asia from 4.95°N to 60.05°N and 64.75°E to 150.25°E (Fig. 1). In order to construct the bias correction and a UNet downscaling model, the high-resolution MSWX datasets are averaged to coarse resolution at 1.0° × 1.0° as MSWX_LR using the area average method.

For climate change downscaling, we use the CMIP6 data, which provides the latest GCM simulations including voluminous global gridded model data over the historical period of 1950–2014 and four Shared Socioeconomic Pathways (SSPs) scenarios with 2015–2100 period. There are 19 GCM outputs for historical simulations and three representative future scenarios (SSP1-2.6, SSP2-4.5, and SSP5-8.5) (Table 2). As shown in Table 2, the original CMIP6 GCMs outputs have coarse spacing resolution. All CMIP6 data can be downloaded at https://esgf-node.llnl.gov/projects/cmip6/.

**Table 2:** CMIP6 modes included in downscaled archive.

Model	Variant	Resolution	tas	tasmax	tasmin	pr	sfcWind	rlds	rsds	hurs	huss
ACCESS-ESM1-5^57,58^	r1i1p1f1	1.875° × 1.25°	O	O	O	O	O	O	O	X	X
BCC-CSM2-MR^59^	r1i1p1f1	1.125° × 1.125°	O	O	O	O	O	O	O	X	O
CanESM5^60^	r1i1p1f1	2.81° × 2.81°	O	O	O	O	O	O	O	O	O
CESM2^61^	r1i1p1f1	1.25° × 0.94°	O	X	X	O	X	O	O	X	O
CMCC-ESM2^62^	r1i1p1f1	1.25° × 0.94°	X	X	X	X	O	O	O	O	O
CNRM-CM6-1^63^	r1i1p1f2	1.41° × 1.41°	O	O	O	O	X	O	O	O	O
CNRM-ESM2-1^64^	r1i1p1f2	1.41° × 1.41°	O	O	O	X	X	O	O	O	X
EC-Earth3^65^	r1i1p1f1	0.70° × 0.70°	O	O	O	O	O	O	O	O	O
FGOALS-g3^66^	r1i1p1f1	2° × 2.25°	O	O	O	O	O	O	O	O	O
GFDL-ESM4^67^	r1i1p1f1	1.25° × 1.0°	O	O	O	O	O	O	O	O	O
INM-CM5-0^68,69^	r1i1p1f1	2° × 1.5°	O	O	O	O	O	O	O	O	O
IPSL-CM6A-LR^70^	r1i1p1f1	2.5° × 1.26°	O	O	O	O	O	O	O	O	X
MIROC6^71^	r1i1p1f1	1.41° × 1.41°	O	O	O	O	O	O	O	X	X
MIROC-ES2L^72^	r1i1p1f2	2.81° × 2.81°	O	O	O	O	X	X	X	X	O
MPI-ESM1-2-HR^73,74^	r1i1p1f1	0.94° × 0.94°	O	O	O	O	O	O	O	O	O
MPI-ESM1-2-LR^73^	r1i1p1f1	1.875° × 1.875°	O	O	O	X	O	O	O	O	O
MRI-ESM-2.0^75^	r1i1p1f1	1.125° × 1.125°	O	O	O	O	O	O	O	O	O
NorESM2-LM^76^	r1i1p1f1	2.5° × 1.875°	O	O	O	O	O	O	O	O	O
NorESM2-MM^76^	r1i1p1f1	1.25° × 0.94°	O	O	O	O	O	O	O	O	O

O means all experiments available (historical, SSP1-2.6, SSP2-4.5, SSP5-8.5); X means no data available.

### BC-UNet

**Fig. 2:**
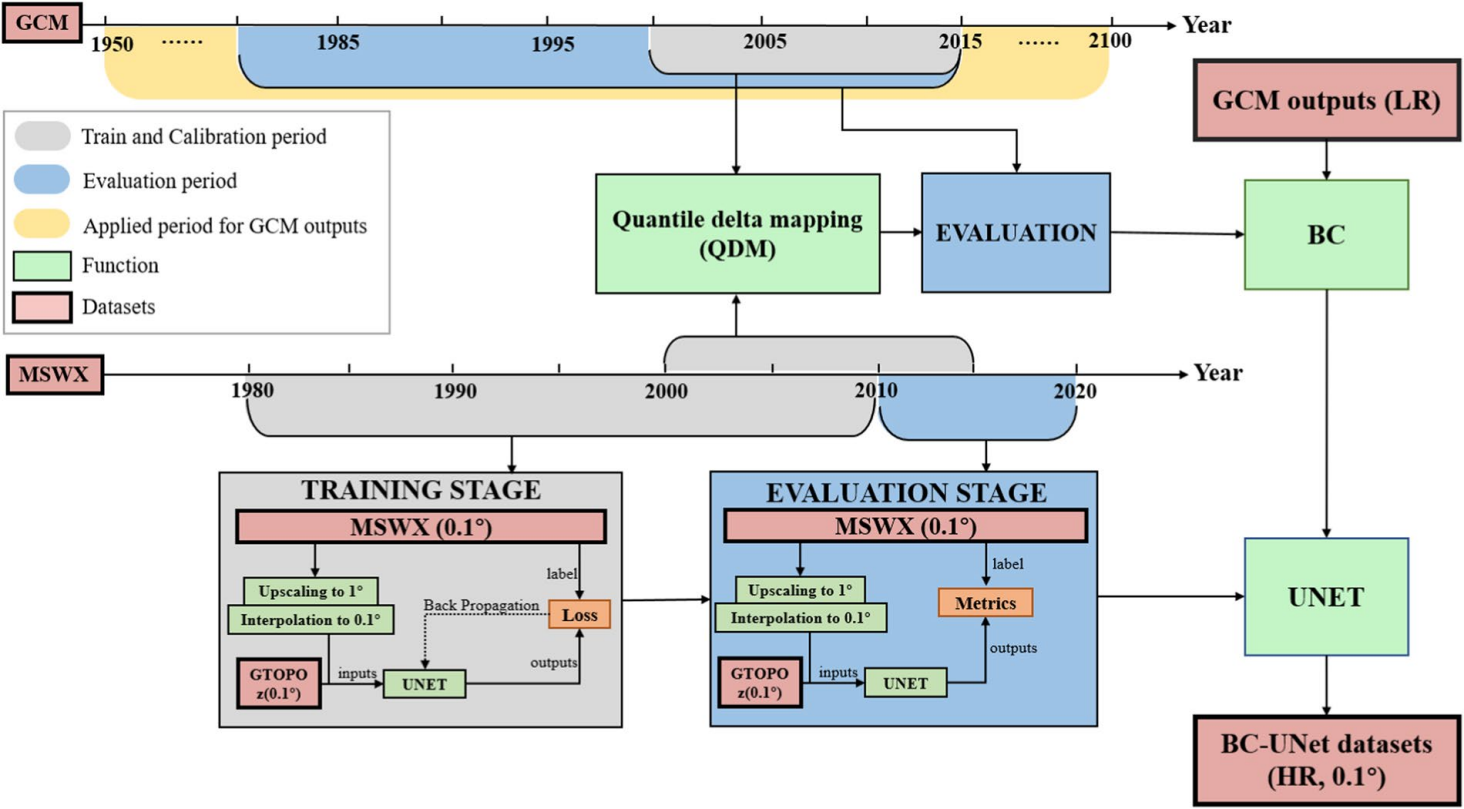
BC-UNet framework flow chart.

The framework to construct the CLIMEA-BCUD, called BC-UNet is demonstrated in Fig. 2. BC-UNet takes GCM simulation datasets and observation as input. It has two main steps: (1) bias correction and (2) UNet downscaling. The details of the two steps are as below.

In the first step, the bias correction method using QDM is applied, which can reduce the bias between observations and GCMs outputs and preserves the change of model projection in quantile^39,40^. When applying bias correction, the GCMs outputs are interpolated to 1° × 1° coarse horizontal resolution to match the MSWX_LR with bi-linear interpolation algorithm. Then QDM is used to correct the biases between GCMs and MSWX_LR at coarse resolution, and to calculate the bias corrected GCM results (GCM_BC).

**Fig. 3:**
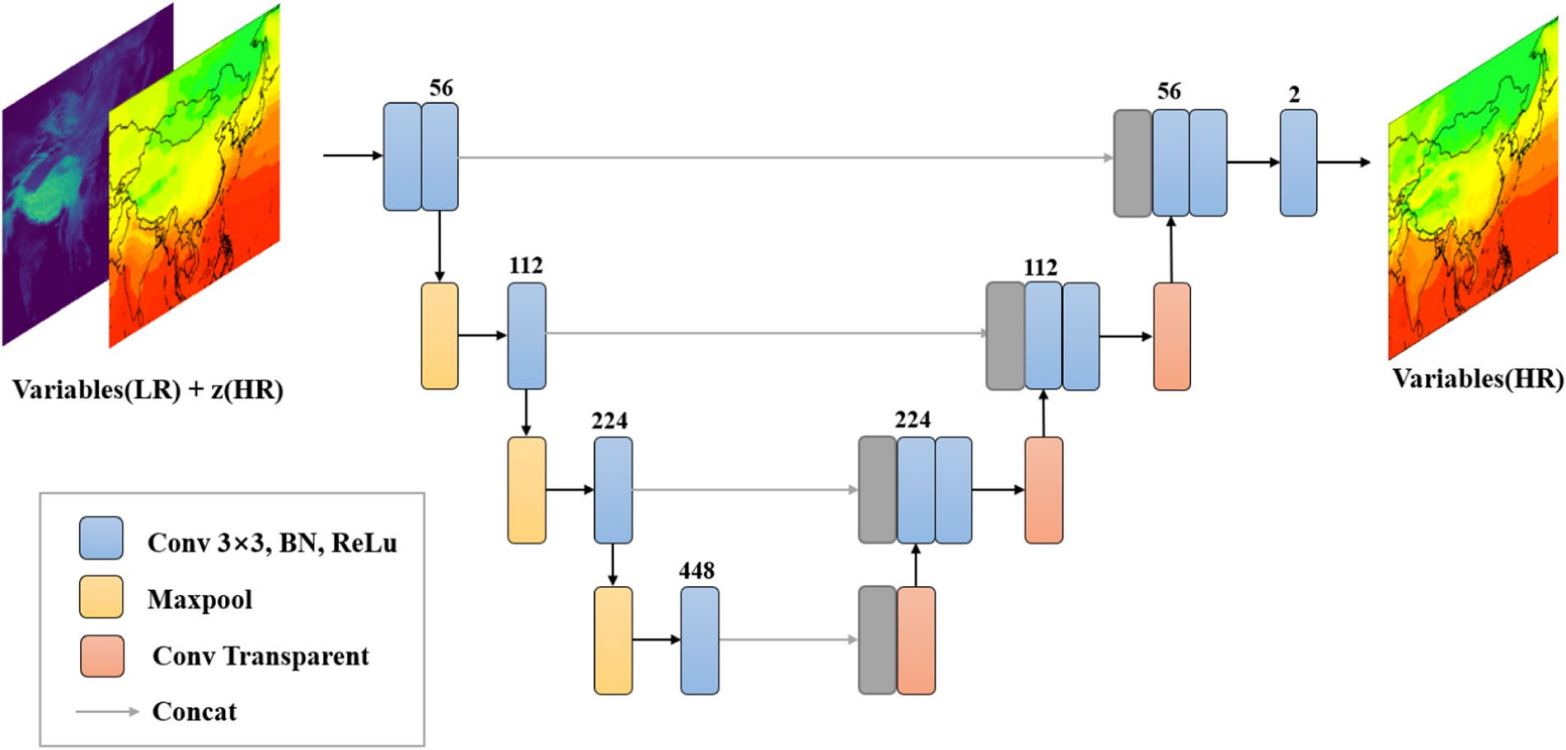
UNet framework in this study. Blue block indicates the convolution, batch normalization and ReLu operation. Yellow block corresponds to the max pooling operation to downsampling. Red block means the transparent convolution operation to upsampling. Grey arrow and grey block means the skip connection to merge the feature maps.

In the second step, UNet with 3 layers neural network, known for its exceptional performance in super-resolution and downscaling tasks, is used for climate downscaling^41^. Every convolution and downsampling operation lead to a feature map, which captures the spatial features. The UNet with 3 layers represent 3 downsampling and 3 upsampling. A convolution operation of each layer will generate a feature map, and the number of convolution channels represents the number of feature maps extracted by this layer. The downsampling component of UNet captures crucial spatial features, while the upsampling counterpart generates high-resolution data, effectively facilitating the downscaling process. The convolution channel numbers to capture the spatial features in UNet are {64, 96, 128, 160} for precipitation and {56, 112, 224, 448} for the other variables (Fig. 3).

As the goal of training stage, the loss function^42^ plays an important role in directing the neural network parameter update. The neural network minimizes the loss function value by continuously updating its parameters during the training stage. Training of the UNet model is completed when the loss function converges to the minimum. This study proposes a new loss function based on the mean absolute error (MAE). The loss function effectively augments the UNet’s capacity to regenerate extreme precipitation events and mitigating the bias of variable underestimation. The loss function is as follows:

$$Loss=\frac{1}{n}\mathop{\sum }\limits_{i=0}^{n}\left|{y}_{p,i}-{y}_{o,i}\right|+\frac{w}{m}\mathop{\sum }\limits_{j=0}^{m}\left|{y}_{p,j}-{y}_{o,j}\right|$$

Where *i* is the grid point which are less than mean, and *j* indicates grid point which are greater than mean. Weight *w* is 5 to decrease the underestimation of downscaling model.

In order to effectively capture the fine features of the MSWX dataset in different seasons, four UNets are trained for each variable, with each UNet being responsible for a different season (MAM, JJA, SON and DJF for spring, summer, autumn and winter respectively). To achieve this, inputs for each season are constructed from data (0.1° × 0.1°) which is downscaled from MSWX_LR by a factor of 10 using bi-linear interpolation algorithm and static elevation (z; coarse to 0.1° spacing resolution) from Global 30 Arc-Second Elevation^43^ (GTOPO30), and original MSWX serves as label for each season. This study feed the univariate image and terrain data to the UNet, and the outcome is a single image. The UNet uses max-pooling for downsampling, deconvolution for upsampling, and long-hop connections to concatenate feature maps of the same resolution. All inputs and labels are spatially normalized before being fed into the UNet. Adaptive moment estimation (Adam) are used as the optimizer in optimization process during the training stage. The GCM_BC are normalized and fed into the trained UNet to generate the downscaling results. Finally, the downscaling results are denormalized to generate CLIMEA-BCUD.

## Data Records

CLIMEA-BCUD contains nine meteorological variables (Table 1) of about 19 downscaled CMIP6 outputs. It has the spatial coverage of 4.95°N–60.05°N and 64.75°E–150.25°E at 0.1° × 0.1° horizontal resolution. The time period of historical climate ranges from January 1, 1950 to December 31, 2014. The future period of three climate change scenarios (SSP1-2.6, SSP2-4.5, SSP5-8.5) is from January 1, 2015 to December 31, 2100 at daily intervals. All data are archived in the NetCDF format in CLIMEA-BCUD, named as “/{variables}/{scenarios}/{year}.nc”, where {variables} is the name of the variables, {scenarios} refers to the historical and three future scenarios (SSP1-2.6, SSP2-4.5, SSP5-8.5), and {year} is the year, respectively. The size of multi-model ensemble mean data is about 2.0 TB. Due to the large size of the dataset, the Science Data Bank (https://www.scidb.cn/en) is chosen for the dissemination of the multi-model ensemble mean CLIMEA-BCUD (10.57760/sciencedb.07718)^44^.

## Technical Validation

In order to comprehensively assess the accuracy of the CLIMEA-BCUD, the spatial distribution of climate mean, the variation of annual mean and root mean square error (RMSE) are calculated against the MSWX dataset from 1979 to 2014 (Table 3). The RMSEs between the raw GCM and MSWX are listed to assess the accuracy of the CLIMEA-BCUD. Notably, INM-CM5-0, MPI-ESM1-2-HR, and MPI-ESM1-2-LR in CLIMEA-BCUD exhibit better skills with relatively low RMSEs for surface air temperature. Tasmax in CLIMEA-BCUD shows the best performance with the RMSEs below 0.58 °C and MBs between −0.52 °C and −0.27 °C, which is better than the raw GCM with the RMSEs above 2.31 °C and MBs between −1.27 °C and 1.13 °C. Tasmin in CLIMEA-BCUD shows a lower RMSE (0.78 °C) than the raw GCM whose lowest RMSE is 2.32 °C. For precipitation, most CMIP6 models in CLIMEA-BCUD are able to reproduce the distribution of mean precipitation with the RMSEs below 0.37 mm/day, showing better performance than the raw GCM with the RMSEs of around 1.00 mm/day. The surface wind speed in CLIMEA-BCUD has RMSEs ranging from 0.13 m/s to 0.15 m/s and surface relative humidity has a degree of RMSEs between 0.97% and 1.60%. Compared with CLIMEA-BCUD, the surface wind speed in raw GCM has RMSEs ranging from 0.92 m/s to 1.41 m/s and surface relative humidity has a degree of RMSEs between 6.50% and 11.87%. CLIMEA-BCUD also has RMSEs larger than 3.0 W/m^2^ for surface downward radiative fluxes, especially for surface downward longwave radiation.

**Table 3:** RMSEs between MSWX and GCM and CLIMEA-BCUD.

		tas	tasmax	tasmin	pr	sfcWind	rlds	rsds	hurs	huss
ACCESS-ESM1-5	GCM	2.69	2.77	3.03	0.38	1.07	15.66	18.32		
	BCUD	0.56	0.5	0.62	0.29	0.15	4.21	2.69		
BCC-CSM2-MR	GCM	2.83	2.83	3.35	1.18	1.41	11.77	18.43		0.00137
	BCUD	0.47	0.4	0.55	0.27	0.15	3.51	2.7		0.0003
CanESM5	GCM	3.09	2.79	4.10	1.26	1.13	14.87	18.79	11.87	0.00288
	BCUD	0.66	0.58	0.78	0.29	0.14	4.59	3.05	1.14	0.0004
CESM2	GCM	2.44			1.19		13.90	17.11		0.00117
	BCUD	0.59			0.29		4.17	2.71		0.00036
CMCC-ESM2	GCM					1.20	13.79	16.98	9.37	0.00112
	BCUD					0.15	3.73	2.71	1.14	0.00035
CNRM-CM6-1	GCM	2.95	2.64	3.49	1.10		13.01	23.15	7.25	0.00163
	BCUD	0.42	0.36	0.5	0.27		3.06	2.7	0.97	0.00025
CNRM-ESM2-1	GCM	2.68	2.49	3.11			11.36	23.47	7.41	
	BCUD	0.46	0.4	0.53			3.35	2.98	1.08	
EC-Earth3	GCM	2.19	2.31	2.73	0.89	1.08	10.89	18.02	6.50	0.00145
	BCUD	0.62	0.52	0.73	0.29	0.14	4.98	3.3	1.60	0.00043
FGOALS-g3	GCM	3.20	3.49	3.50	1.81	1.39	14.36	25.16	10.52	0.00148
	BCUD	0.59	0.5	0.68	0.28	0.14	4.19	2.83	1.01	0.00032
GFDL-ESM4	GCM	2.23	2.35	2.57	1.15	1.15	10.16	14.84	8.07	0.00113
	BCUD	0.49	0.43	0.56	0.25	0.14	3.51	2.8	1.15	0.00031
INM-CM5-0	GCM	2.61	2.82	2.97	1.47	1.20	13.75	27.14	8.10	0.00143
	BCUD	0.42	0.36	0.51	0.27	0.15	3.18	3.12	1.10	0.00027
IPSL-CM6A-LR	GCM	3.72	3.58	4.42	1.31	1.00	16.65	24.21	11.20	
	BCUD	0.56	0.46	0.66	0.27	0.15	3.77	2.74	0.99	
MIROC6	GCM	2.88	3.92	2.59	1.15	1.34	11.95	19.53		
	BCUD	0.47	0.40	0.55	0.27	0.14	3.84	3.18		
MIROC-ES2L	GCM	2.93	3.20	2.59	1.21					0.00122
	BCUD	0.55	0.51	0.59	0.26					0.00025
MPI-ESM1-2-HR	GCM	2.23	2.37	2.24	1.32	0.92	12.85	20.62	10.47	0.00124
	BCUD	0.43	0.36	0.52	0.29	0.15	3.25	3.09	1.15	0.00028
MPI-ESM1-2-LR	GCM	2.61	2.75	2.65		1.13	16.31	19.45	11.81	0.00145
	BCUD	0.4	0.33	0.49		0.14	3.15	2.94	1.12	0.00028
MRI-ESM2-0	GCM	2.21	2.32	2.32	1.21	1.50	11.01	21.99	8.93	0.00131
	BCUD	0.44	0.36	0.53	0.25	0.14	3.27	3.2	1.07	0.00027
NorESM2-LM	GCM	2.87	2.90	3.05	1.08	1.04	16.03	19.50	8.94	0.00131
	BCUD	0.55	0.49	0.61	0.30	0.13	3.91	2.98	1.05	0.00034
NorESM2-MM	GCM	2.22	2.34	2.32	0.96	0.94	11.06	19.52	7.68	0.00107
	BCUD	0.44	0.38	0.51	0.37	0.15	3.38	3.75	1.03	0.0003

**Fig. 4:**
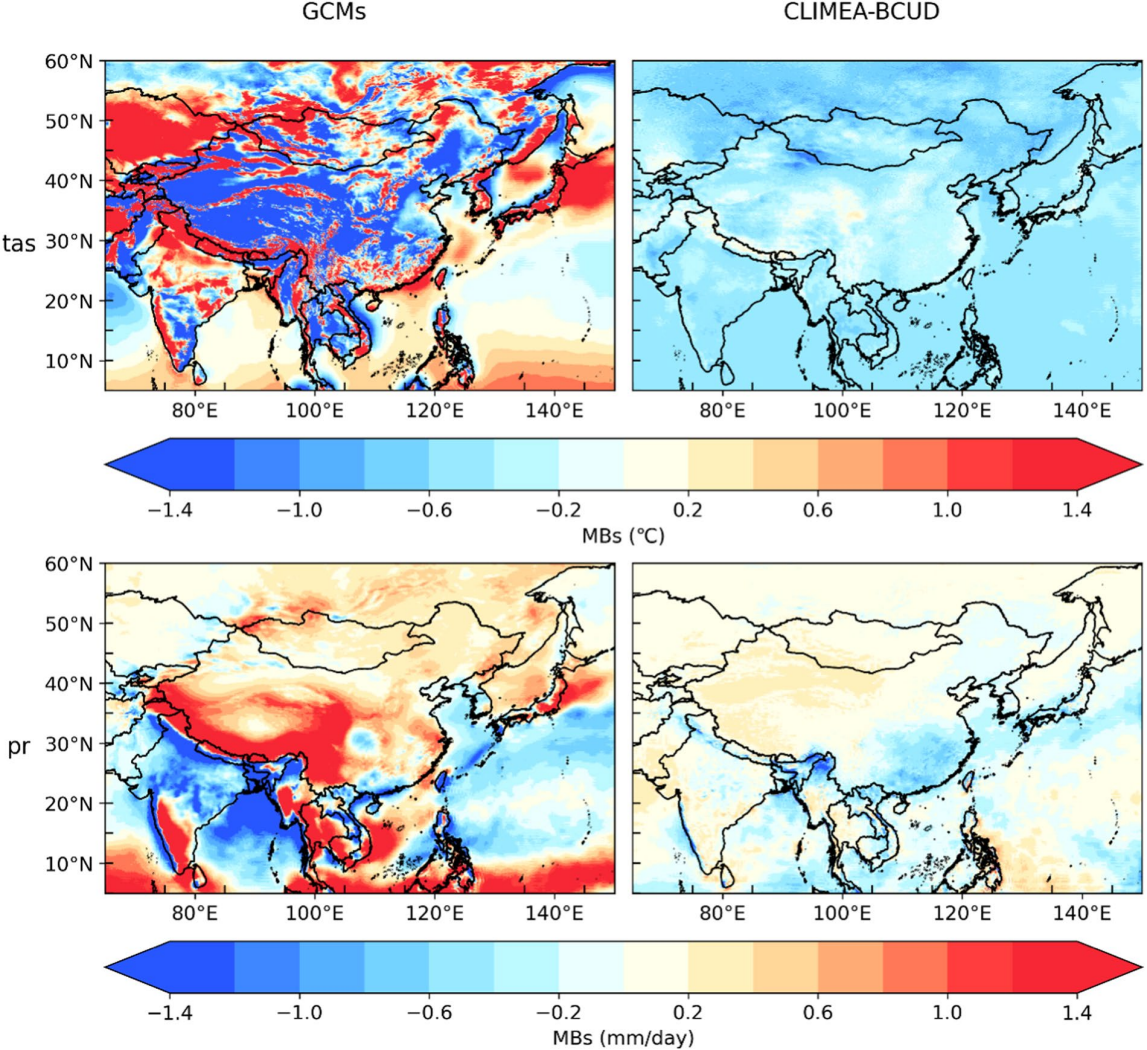
MB distribution of tas and precipitation in CLIMEA-BCUD.

Figure 4 illustrates the distribution of multi-model ensemble mean bias between the raw GCM and MSWX, CLIMEA-BCUD and MSWX. Evidently, tas in CLIMEA-BCUD is comparable to that in MSWX over regions with flat terrain, showing much better performance than the raw GCM which has much larger bias. Even in the high-altitude regions such as the Qinghai-Tibet Plateau, the multi-model ensemble mean of CLIMEA-BCUD is able to capture the key features including the variation of annual mean tas and spatial patterns of the tas climate mean. Compared with CLIMEA-BCUD, tas in the raw GCM is significantly underestimated over the Qinghai-Tibet Plateau. For precipitation, the bias of multi-model ensemble mean ranges from −0.6 mm/day to 0.6 mm/day over most regions in East Asia. While precipitation in the raw GCM is significantly overestimated over East Asia by around 1.0 mm/day. A relatively large bias for CLIMEA-BCUD above 1.0 mm/day can be found over the south eastern side of the Qinghai-Tibet Plateau, and the west coast of Africa, which is smaller than the raw GCM with a bias above 1.4 mm/day. Compared with the raw GCM, CLIMEA-BCUD for all variables can effectively and generally reproduce the spatial distribution of climatological average from 1979 to 2014 with higher SCCs and lower MBs and variation of annual mean with much lower RMSEs.

### Seasonality

**Fig. 5:**
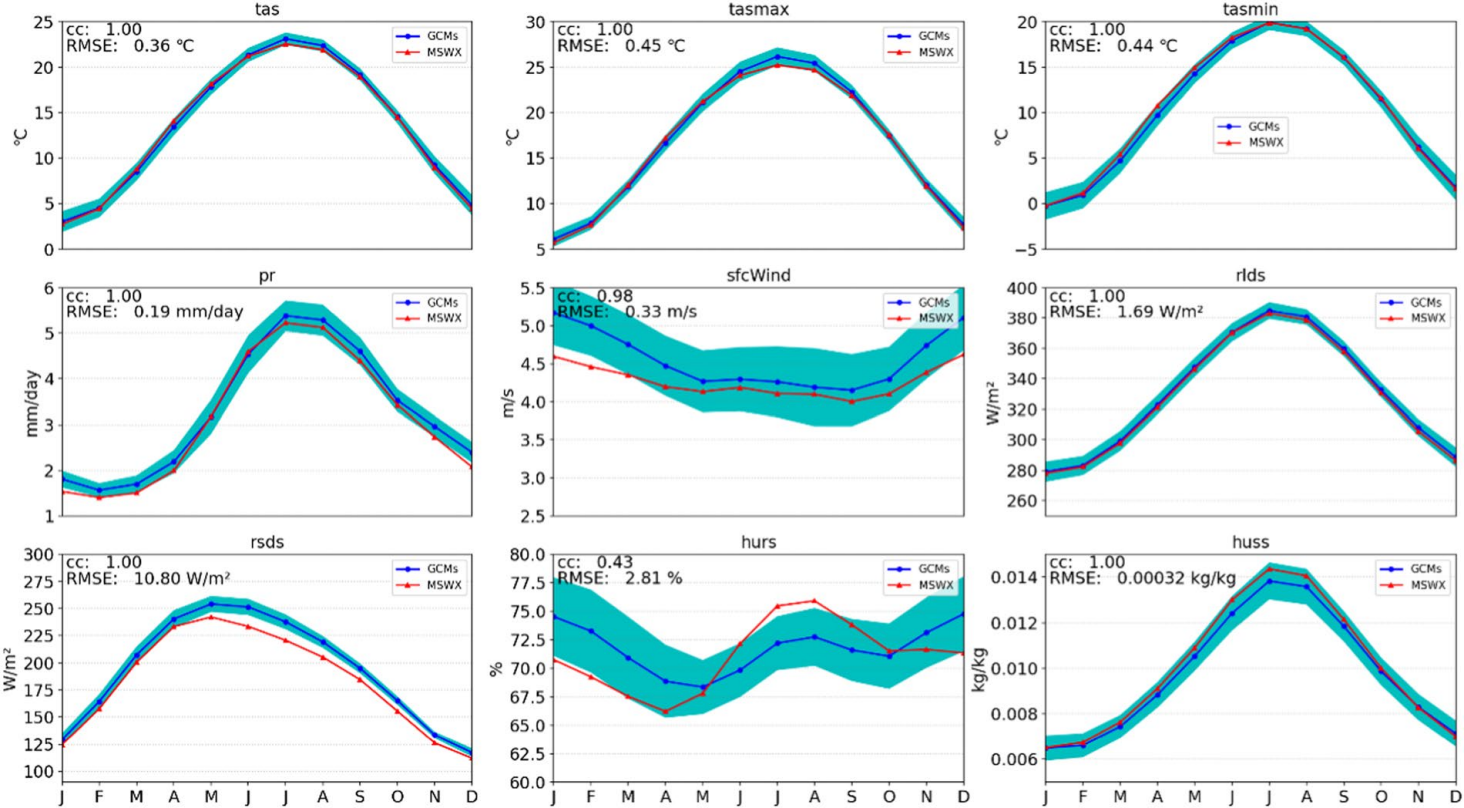
Seasonal cycles of the nine variables during 1979–2014. Red line is the MSWX seasonal cycle. Blue line is the multi-model ensemble mean seasonal cycle of GCMs, the shaded area represents uncertainties of all models with one standard deviation.

**Fig. 6:**
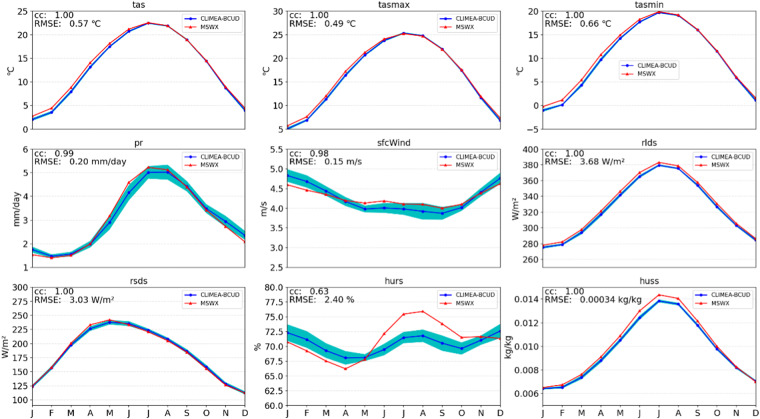
Seasonal cycles of the nine variables during 1979–2014. Red line is the MSWX seasonal cycle. Blue line is the multi-model ensemble mean seasonal cycle of CLIMEA-BCUD, the shaded area represents uncertainties of all models with one standard deviation.

Figure 5 illustrates the seasonal cycle of all variables from the raw GCM output. Figure 6 shows that CLIMEA-BCUD can well reproduce the seasonal cycle of surface air temperature with a correlation of 1.0, but shows large uncertainties and warm biases in summer. Compared with raw GCM, the multi-model ensemble mean of CLIMEA-BCUD can well reproduce the seasonal cycle of surface air temperature with a correlation of 1.0 and lower uncertainties; yet significant cold biases are found in spring and winter. Because of the normalization, surface air temperature in CLIMEA-BCUD maintains the advantage of QDM outputs, which can represent time series with higher CC and lower uncertainties than the raw GCM. For the seasonal cycle of precipitation, the multi-model ensemble mean of CLIMEA-BCUD exhibits a good correlation (0.99) and a low RMSE of 0.2 mm/day, but has relatively large uncertainties, particularly in summer when precipitation shows strong spatio-temporal variability.

Surface wind speed in the multi-model ensemble mean of the raw GCM shows good correspondence with MSWX with a high correlation of 0.98 and RMSE of 0.33 m/s, but it clearly overestimates wind speed and exhibits a large uncertainty. While surface wind speed in the multi-model ensemble mean of CLIMEA-BCUD shows good coherence with MSWX with a high correlation of 0.98 and a lower RMSE of 0.15 m/s and significantly reduces the uncertainty, it clearly overestimates wind speed in winter and underestimates it in summer. The surface relative humidity displays a lower degree of seasonal variation than that of MSWX, leading to a rather low correlation of 0.63, which is still higher than the raw GCM (correlation 0.43). Multi-model ensemble mean of CLIMEA-BCUD can well generate downward longwave radiation, downward shortwave radiation, and surface specific humidity. In general, the multi-model ensemble mean of CLIMEA-BCUD, compared with the raw GCM, reduces the uncertainties and achieves higher correlation and lower RMSE.

### Extreme events

**Fig. 7:**
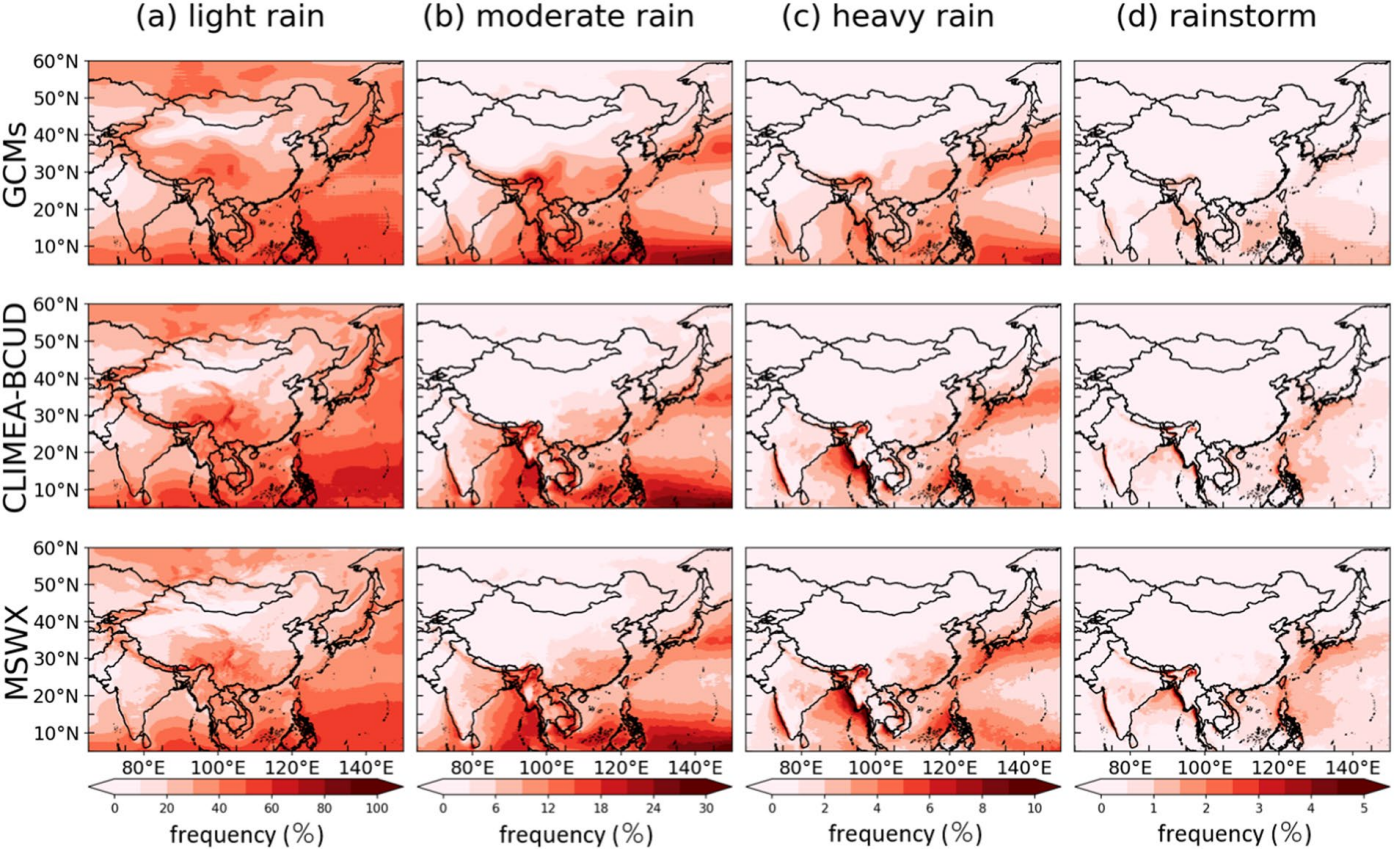
Frequency of different rank (a for light rain, b for moderate rain, c for heavy rain and d for rainstorm) of precipitation in GCMs, CLIMEA-BCUD and MSWX.

Regarding the precipitation events, 4 distinctive classes of precipitation events are categorized: light rain (1 ≤ pr < 10 mm/day), moderate rain (10 ≤ pr < 25 mm/day), heavy rain (25 ≤ pr < 50 mm/day) and rainstorm (pr ≥ 50 mm/day) according to the China Meteorological Administration^45^ (CMA). By counting the frequency of precipitation events at each grid and comparing it with the raw GCM, the performance of the CLIMEA-BCUD in generating the precipitation events can be assessed (Fig. 7). For the light rain events, CLIMEA-BCUD is capable of capturing the overall pattern of MSWX, and shows more detail than the raw GCM. QDM can preserve daily precipitation extreme events well, which are also preserved by CLIMEA-BCUD. CLIMEA-BCUD has a higher frequency between 60% and 70% than MSWX which is below 60% over the eastern Pacific. For moderate rain events, the over shift of rain belt for the raw GCM is found in the eastern Pacific and the Qinghai-Tibet Plateau. Moreover, the raw GCM overestimates the frequency over southern China. CLIMEA-BCUD performs better in producing the distribution of frequency, with two main rain belts over the Pacific. But it slightly underestimates the frequency over land areas, especially over southeastern China. For the heavy rain events, GCMs overestimate the frequency over the southeastern Pacific and southern China. CLIMEA-BCUD can capture the spatial distribution of frequency with slight underestimation over most regions in East Asia and perform more details than the raw GCM. For the rainstorm events, the raw GCM cannot regenerate the distribution over East Asia. CLIMEA-BCUD can reproduce the distribution over oceanic areas. Notably, CLIMEA-BCUD narrows down areas with rainstorm events frequency between 1% and 2%, especially over the Kyushu region of Japan. In general, CLIMEA-BCUD can capture different rank precipitation events well, especially moderate rain, but there are some obvious biases in the eastern Pacific.

### Projected changes

**Fig. 8:**
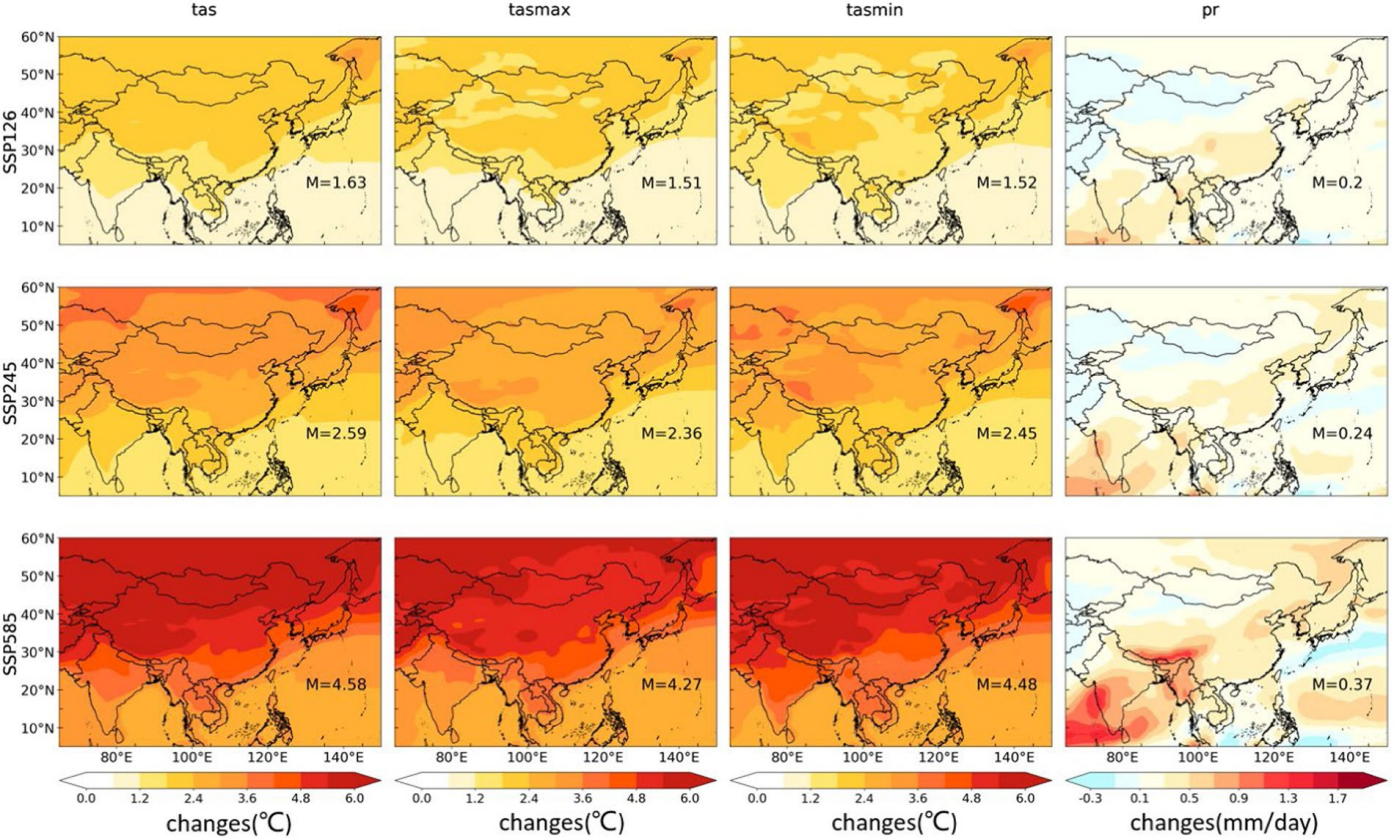
Future change of surface air temperature and precipitation in the end 21^st^ century (2070–2100) from GCMs.

**Fig. 9:**
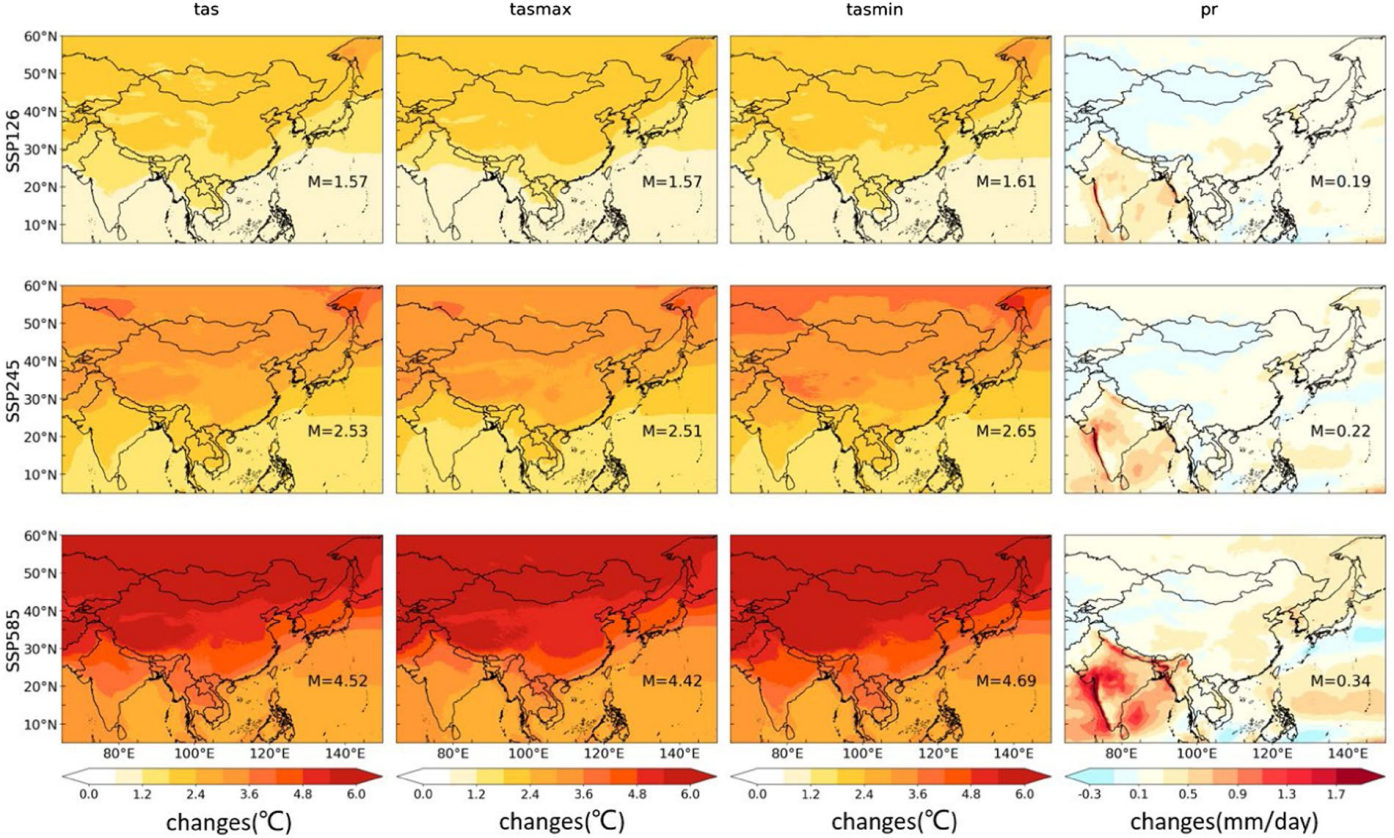
Future change of surface air temperature and precipitation in the end 21^st^ century (2070–2100) from CLIMEA-BCUD.

Based on the evaluation of downscaled daily precipitation and surface air temperature, projections in surface air temperature and precipitation at the end of the 21st century (2070–2100) from CLIMEA-BCUD for all the scenarios (SSP1-2.6, SSP2-4.5, and SSP5-8.5) can be estimated. Figure 8 (the raw GCM) and Figure 9 (CLIMEA-BCUD) shows the changes in multi-model ensemble mean surface air temperature and precipitation at the end 21^st^ century for all the scenarios (SSP1-2.6, SSP2-4.5, and SSP5-8.5). It is found that the surface air temperature will rise in East Asia, with a greater warming range in the northern part of China especially under the SSP5-8.5 scenario, which shows a similar distribution with the raw GCM. The ensemble mean median change in tas from CLIMEA-BCUD is projected to increase by 1.57 °C in SSP1-2.6, 2.53 °C in SSP2-4.5, and 4.52 °C in SSP5-8.5, which is similar to the raw GCM with 1.63 °C in SSP1-2.6, 2.59 °C in SSP2-4.5 and 4.58 °C in SSP5-8.5. The projection of ensemble mean tasmax and tasmin from CLIMEA-BCUD is similar to that of tas, with the temperature increasing from south to north across East Asia, indicating that the CLIMEA-BCUD preserves the climatic trend from the raw GCM. In terms of precipitation, the projected change in CLIMEA-BCUD generally shows an increase over most areas in East Asia, and the ensemble mean median change is projected to increase by 0.19 mm/day in SSP1-2.6, 0.22 mm/day in SSP2-4.5 and 0.34 mm/day in SSP5-8.5. While the projected change in the raw GCM has the same changes and the ensemble mean median change is projected to increase by 0.20 mm/day in SSP1-2.6, 0.24 mm/day in SSP2-4.5 and 0.37 mm/day in SSP5-8.5. A significant increase of precipitation in the raw GCM is found over the Indian Ocean and the western Pacific Ocean. For CLIMEA-BCUD, precipitation will significantly increase in eastern China, and slightly decrease in the northwestern regions. It will also increase in India, especially under the SSP5-8.5 scenario. The increase of precipitation over the ocean is more notable, mainly in the Indian Ocean and the western Pacific Ocean.

## Usage Notes

In this study, we describe the CLIMEA-BCUD dataset for East Asia, which provides daily time series of nine meteorological variables at 0.1 spacing resolution based on 19 CMIP6 GCMs. CLIMEA-BCUD is provided for both the historical period (1950–2014) and the future period (2015–2100), and it incorporates three different emission scenarios for the future: SSP1-2.6, SSP2-4.5 and SSP5-8.5. By delivering such high-resolution information, CLIMEA-BCUD can be very useful for various hydroclimatic research. Furthermore, CLIMEA-BCUD may also prove useful for users not only in the hydrometeorological field but also in others, such as climate change, agriculture, energy, etc. Given East Asia’s continental proportions and its role in global climate, the high resolution (0.1°) of gridded data is critical for developing regional and global assessments and aiding decision- and policy-making. CLIMEA-BCUD is presented in netCDF format (.nc), and it is freely available at the Science Data Bank (10.57760/sciencedb.07718)^44^. While CLIMEA-BCUD has a wonderful performance in producing the overall patterns of climate mean, seasonal cycle, frequency, and future changes, some limitations must be acknowledged. Firstly, data users should be aware of underestimation when using CLIMEA-BCUD due to its underestimation in representing observations. Secondly, despite displaying good performance in reproducing seasonal variability and extreme events, the bias-corrected products may contain inherent uncertainties, and obscure some fundamental deficiencies presented by the climate models.

Numerous studies have extensively researched methods to enhance model performance in the field of super-resolution, and these advancements are expected to be applicable to downscaling tasks as well. Among them, image enhancement techniques including adaptive gamma correction with weighting distribution^46^ (AGCWD), adaptive gamma correction with color preserving framework^47^ (AGCCPF), range limited Bi-histogram equalization^48,49^ (RLBHE), and region adaptive contrast limited adapted histogram equalization^50^ (RACLAHE) are common and powerful tools for improving the performance of DL model. It is valuable to explore its effectiveness in the context of climate downscaling. Furthermore, several studies have explored improved models based on UNet such as UNet++^51^, UNet3+^52^, ResUNet^53^ and USE-NET^54^, which have demonstrated significant potential in various applications. Additionally, models that combine technologies such as generative adversarial network^55^ (GAN) and Transformer^56^ have also shown great potential for further improvement.

## Data Availability

QDM approach in this study is carried out using the R-packages of the Multivariate Bias Correction of Climate Model Outputs (MBC) project and it is available through the following Github link: https://github.com/cran/MBC. The UNet downscaling approach is carried out using the python-packages of the tensorflow2 and it is available through the following Github link: https://github.com/tensorflow/tensorflow. All code used in this study can be available through the following Github link: https://github.com/LinHai-debug/CLIMEA-BCUD-code.
